# Identifying Crude Drugs in Kampo Medicines Associated with Drug-Induced Liver Injury Using the Japanese Adverse Drug Event Report Database: A Comprehensive Survey

**DOI:** 10.3390/ph16050678

**Published:** 2023-05-01

**Authors:** Kyosuke Kimura, Mami Kikegawa, Yusuke Kan, Yoshihiro Uesawa

**Affiliations:** 1 Department of Medical Molecular Informatics, Meiji Pharmaceutical University, Kiyose 204-8588, Japan; 2 Datack, Inc., Tokyo 102-0072, Japan; 3 Department of Kampo Medicine, Yokohama University of Pharmacy, Yokohama 245-0066, Japan; 4 Nanohana Pharmacy, Tomakomai 053-0021, Japan

**Keywords:** drug-induced liver injury (DILI), Kampo medicine, crude drug, spontaneous reporting system, Japanese Adverse Drug Event Report (JADER) database, pharmacovigilance, volcano plot, reporting odds ratio (ROR)

## Abstract

The current study aimed to identify the crude drugs associated with drug-induced liver injury (DILI) in 148 Kampo medicines prescribed throughout Japan using the Japanese Adverse Drug Event Report (JADER) database, a large-scale spontaneous reporting system in Japan. First, we tabulated the number of DILI reports from the report-based dataset and the background information from the patient-based dataset. Thereafter, we combined the 126 crude drugs into 104 crude drug groups to examine multicollinearity. Finally, the reporting odds ratios (RORs), 95% confidence intervals, *p* values for Fisher’s exact test, and number of reports were calculated for each crude group to identify those associated with DILI. Notably, the number of adverse event reports for DILI (63,955) exceeded that for interstitial lung disease (51,347), the most common adverse event. In total, 78 crude drug groups (90 crude drugs) were reported to have an ROR > 1, a *p* < 0.05, and ≥10 reported cases. Our results highlight DILI as an essential issue, given that it was among the most frequently reported adverse drug reactions. We were able to clearly identify the crude drugs associated with DILI, which could help manage adverse drug reactions attributed to Kampo medicines and crude drugs.

## 1. Introduction

Traditional medicines have been used clinically in several parts of the world, including Japan (Kampo medicine), China, Korea, and India (Ayurveda). Generally, traditional medicines are mixtures of multiple herb extracts and contain numerous components derived from medicinal plants. Japanese Kampo medicine originated from Chinese medicine and developed uniquely in Japan. Kampo medicines are, in principle, combinations of several herbal medicines found in nature, such as plants and minerals.

The first Kampo medicines used in modern medical practice were listed in the drug price list of 1967. Around 1980, several formulations were added, leading to an expansion in the number of prescriptions issued throughout Japan. At that time, the efficacy and safety of the medicines were ensured by the long clinical experience of medical practitioners, and the drugs were approved without clinical trials [1]. For quality control, Japan’s Ministry of Health, Labour and Welfare published approval standards for over-the-counter (OTC) Kampo products, which provided information regarding the standard amounts of the constituent crude drugs and the range of indications for which they can be used. Notably, unlike Western medicines, which usually comprise only one ingredient, each Kampo medicine comprises multiple crude drugs, and quality control is conducted based on the evaluations performed in accordance with the abovementioned standards [2].

Currently, 148 types of Kampo preparations are available for use [3]. Crude drugs are often boiled and used as decoctions or dried powders. However, they can also be factory-produced by pharmaceutical companies in Japan and supplied in a ready-to-use form. Over 80% of Japanese physicians prescribe Kampo medicines either as a single agent or in combination with Western medicines [4], establishing Kampo medicines as the primary treatment option in Japan.

Traditionally, Kampo medicines were considered relatively safe with few side effects. Nonetheless, case reports and the Drug and Medical Device Safety Information Reporting System revealed various side effects, which have been attracting increasing attention. The side effects of Kampo medicines can be typically categorized into immunoallergic reactions (e.g., liver injury and interstitial pneumonia caused by Scutellaria Root), overdose (e.g., sympathomimetic symptoms caused by Ephedra Herb, neuroparalysis caused by Processed Aconite Root, and diarrhea caused by Rhubarb), and chronic administration (mesenteric phlebosclerosis caused by Gardenia Fruit) [1]. The suspected cause, the crude drug chemicals involved, and their mechanisms are often clearly identifiable for side effects related to overdose and chronic administration. However, the causes and pathogeneses of immune and allergic reactions remain poorly understood. Several reports noted adverse reactions of liver injury and interstitial pneumonia from formulas containing Scutellaria Root. However, the mechanism by which these adverse reactions develop remains unknown. In addition, aside from Scutellaria Root, other crude drugs were also suggested to be associated with the aforementioned adverse reactions [1,5,6,7].

In particular, the primary treatment for drug-induced liver injury (DILI) is to identify the drug responsible and discontinue its administration as soon as possible [8]. Although mild hepatotoxicity improves spontaneously, some cases may be severe and difficult to treat due to delays in detection or individual differences. Hepatoprotective drugs and corticosteroids can be used for treatment; however, sufficient scientific evidence for this has not been obtained. Therefore, it is essential to identify crude drugs and component chemicals in the Kampo medicines associated with DILI.

Most studies on the adverse effects of Kampo medicine reported to date focused on one type of Kampo medicine or one type of crude drug. This is because Kampo medicines are composed of multiple crude drugs, making their evaluation complicated and challenging. Moreover, given the numerous different types of Kampo medicines and crude drugs, obtaining a complete picture is impractical considering the vast cost and time required to study them. Therefore, an exhaustive analysis is necessary to narrow down the side effects of many Kampo medicines and crude drugs. Unfortunately, such reports are scarce, with only one study by Arai et al. having comprehensively reviewed Japanese Kampo medicines. In the mentioned study, the authors identified crude drugs that contribute to liver injury in only 65 of the 148 Kampo medicines classified as ethical drugs and that had known production values [9]. In other words, an exhaustive study of all Japanese Kampo medicines has not yet been conducted.

To address this issue, the current study used the Japanese Adverse Drug Event Report (JADER) database, which contains many reports of adverse drug events caused by Japanese Kampo medicines [10]. The JADER is a database managed by the Pharmaceuticals and Medical Devices Agency (PMDA) that collects adverse event reports for pharmaceuticals in Japan. This database has been recognized as a valuable tool for evaluating adverse drug reactions. Using the JADER database, we then conducted an exhaustive analysis of the 126 crude drugs contained in all 148 Kampo medicines that fall under the category of ethical drugs to identify the crude drugs associated with DILI.

## 2. Results

### 2.1. Construction of Datasets for Analysis

**Figure 1:**
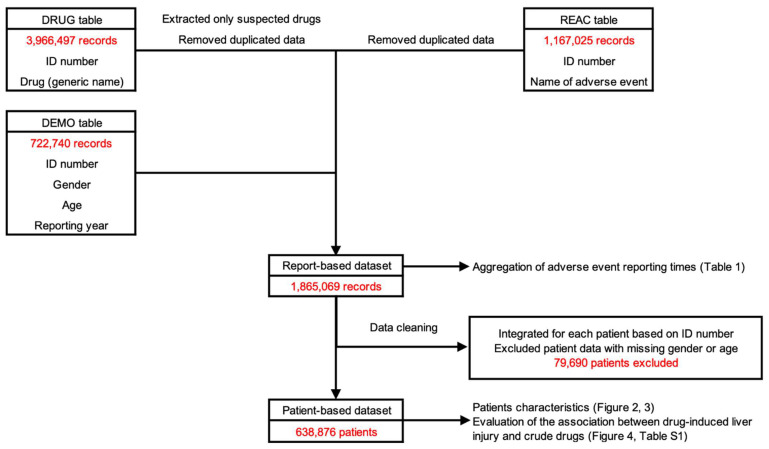
Flow diagram of dataset construction for analysis. The causes of drug-related adverse events (drug name, causal relationship, etc.) for each drug included in the DRUG table were classified into three categories: “suspected drug”, “concomitant drug”, and “interacting drug”. Only “suspected drug” information was extracted from the DRUG table. Duplicate data (adverse events, outcomes, etc.) in the DRUG and REAC tables were deleted. Data from the DEMO table (demographic information such as patient gender, age, weight, etc.) were combined into the DRUG and REAC tables using the patient identification number (ID number). This created a report-based dataset. The report-based dataset was assigned a flag to determine whether the adverse event was DILI, a flag to determine whether the suspected drug was a Kampo medicine, and a flag for the 126 crude drugs contained according to the composition of the Kampo medicines. The dataset was then integrated so that the ID number would be unique. In doing so, priority was given to “applicable” for each assigned flag. Finally, we created a patient-based dataset by excluding cases with missing information on gender and age.

The flow diagram for extracting the dataset from the JADER database is shown in Figure 1. Data were extracted from the drug information (DRUG) table (3,966,497 records), adverse reaction information (REAC) table (1,167,025 records), and patient information (DEMO) table (722,740 records). Data from the three tables were merged to create a report-based dataset (1,865,069 records). In addition, data were merged according to ID number, and ineligible records were deleted to create the patient-based dataset (638,876 records).

### 2.2. Adverse Events Included in the Report-Based Dataset

The report-based dataset was used to determine the number of reported adverse events, with Table 1 summarizing the 50 most frequently reported events. Among the 1,865,069 records, the most frequently reported adverse events were interstitial lung disease (51,347 records), hepatic function abnormal (33,663 records), platelet count decreased (32,904 records), neutrophil count decreased (27,409 records), pyrexia (25,662 records), pneumonia (25,457 records), white blood cell count decreased (25,134 records), anemia (22,024 records), neutropenia (21,246 records), and liver disorder (20,746 records). Among the 50 the most frequently reported adverse events, DILI (hepatic function abnormal, liver disorder, and DILI) accounted for 63,955 reports, which exceeded the number for interstitial lung disease—the most common adverse event.

### 2.3. Characteristics of the Patient-Based Dataset

**Figure 2:**
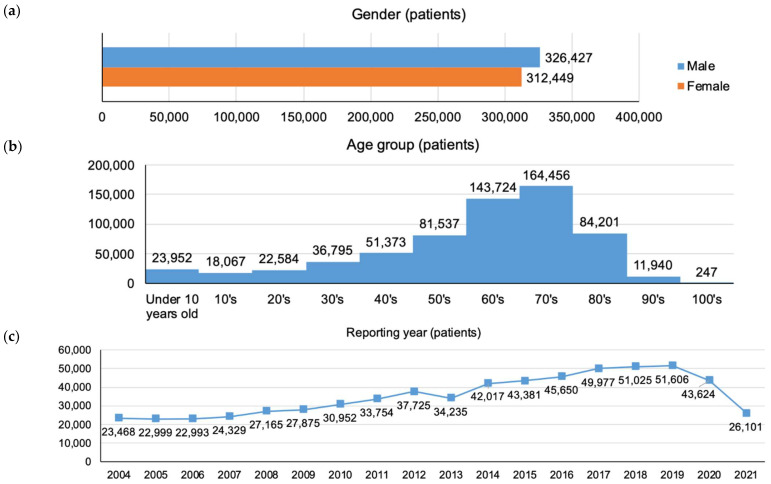

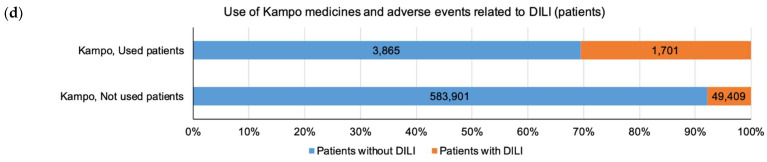
Characteristics of the patient-based dataset (N = 638,876). (**a**) Bar graph for gender; (**b**) histogram for age group; (**c**) line graph for reporting year. The data collection period was from April 2004 to November 2021; thus, the number of reports for FY2021 seems to be low. (**d**) Horizontal bar chart for using Kampo medicines and adverse events related to DILI.

The patient-based dataset was used to obtain information regarding patient background, reporting year, use of Kampo medicines, and DILI-related adverse events (Figure 2). Among the 638,876 patients, men and women were equally represented (326,427, 51.1% vs. 312,449, 48.9%, respectively). Patients in their 60s (143,724, 22.5%) and 70s (164,456, 25.7%) accounted for the largest population in terms of age groups. An increasing trend in the number of reports was found every year since the reporting year. Among the patients with adverse events, 5566 (0.9%) used Kampo medicines. The number of patients with adverse events related to DILI was 51,110 (8.0%). Moreover, the number of patients who used Kampo medicines and experienced adverse events related to DILI was 1701 (0.3%) (Figure 2d).

### 2.4. Characteristics of Patients Who Used Kampo Medicines and Experienced DILI

**Figure 3:**
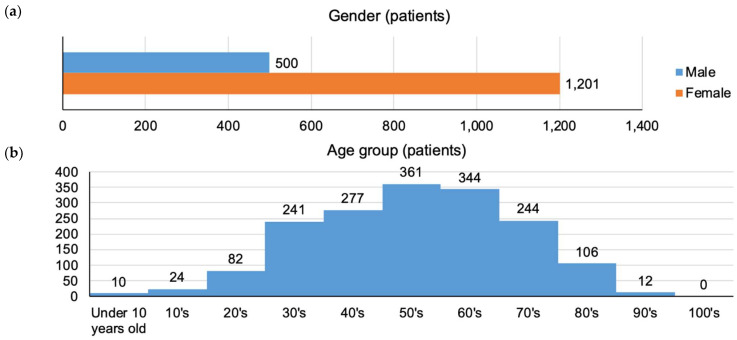

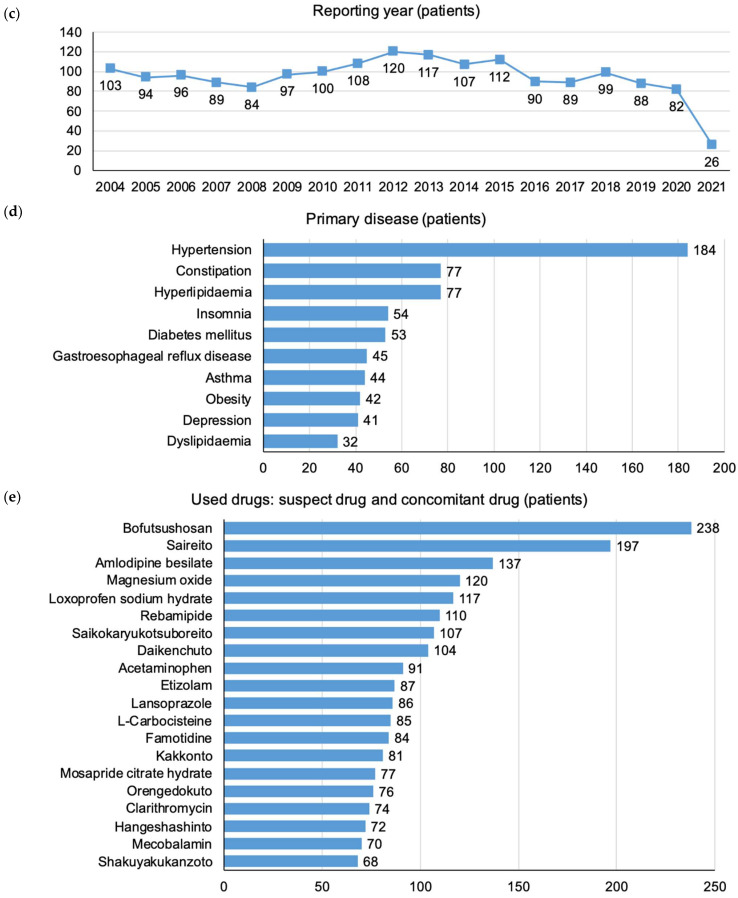
Characteristics of patients who used Kampo medicines and experienced DILI (N = 1701). (**a**) Bar graph for gender; (**b**) histogram for age group; (**c**) line graph for the reporting year. The data collection period was from April 2004 to November 2021; thus, the number of reports for FY2021 seems to be low. (**d**) Ranking of the top 10 primary diseases. The data were obtained from 1055 patients whose primary disease information was available; in JADER, there were patients without primary disease information and patients who were assigned more than once. (**e**) Ranking of the top 20 most-used drugs (suspected and concomitant drugs). As JADER was the source of patient information, data regarding the drugs used were obtained from all 1701 patients; some patients used only one drug, whereas others used multiple drugs.

Focusing on the 1701 patients shown in Figure 2d who used Kampo medicines and experienced DILI, the gender, age, reporting year, primary disease, and drugs used are shown in Figure 3.

The male-to-female ratio of these 1701 patients was approximately 3:7 (males: 29.4%, females: 70.6%). The age groups that were more common were the 50s (21.2%) and 60s (20.2%). The reporting year exhibited a flat trend. Regarding the primary disease information, the 10 most-common diseases were hypertension, constipation, hyperlipidemia, insomnia, diabetes mellitus, gastroesophageal reflux disease, asthma, obesity, depression, and dyslipidemia. Further, regarding the information on the drugs used (suspected and concomitant drugs), the 20 most-common drugs, in order of their frequency of use, were as follows: bofutsushosan, saireito, amlodipine besilate, magnesium oxide, loxoprofen sodium hydrate, rebamipide, saikokaryukotsuboreito, daikenchuto, acetaminophen, etizolam, lansoprazole, L-carbocisteine, famotidine, kakkonto, mosapride citrate hydrate, orengedokuto, clarithromycin, hangeshashinto, mecobalamin, and shakuyakukanzoto.

### 2.5. Association between Crude Drugs Contained in Kampo Medicines and DILI

**Figure 4:**
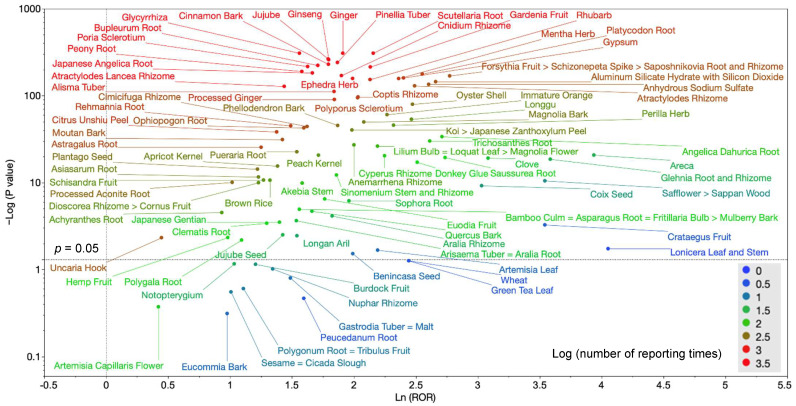
Crude drug groups associated with DILI. The x-axis is the natural logarithm of the reporting odds ratio (Ln (ROR)), whereas the y-axis is the ordinary logarithm of the *p* value from Fisher’s exact test converted to negative (−Log10 (*p* value)). Reporting odds ratios (RORs) were calculated by cross tabulation. The dotted line on the y-axis represents *p* = 0.05. The color of the plot represents the number of adverse events reported. The red–green–blue dots are the ordinary logarithm of the total number of reports (range: 0–3.5); a positive ROR indicates a greater tendency for adverse events to occur, whereas a smaller *p* value indicates a greater statistical significance. Crude drugs having stronger associations with DILI are shown in the upper right-hand corner of the scatterplot.

The scatter plot in Figure 4 shows the association between crude drugs contained in Kampo medicines and DILI. This scatter plot, called a volcano plot, was created using the reporting odds ratio (ROR) and *p* values of all the crude drug groups. Higher vertical axis plots indicate a greater statistical significance, whereas the horizontal axis plots that are more to the right indicate a greater ROR. The tint of the plot also indicates the number of reports, with red dots indicating herbal medicines with a higher number of reports. Therefore, the crude drugs with red dots plotted in the upper right corner are those with the strongest association with DILI.

In addition, by examining the multicollinearity, crude drugs with strong correlations were grouped together in the same group. Thereafter, large or small correlations between the same group of crude drugs and DILI were denoted by “>”, whereas equal correlations were denoted by “=”. The crude drugs with the highest correlation were then integrated to include other crude drugs as representatives.

The crude drug groups with the strongest association with DILI plotted in the upper right corner were: Glycyrrhiza, Ginger, Scutellaria Root, Poria Sclerotium, Jujube, Peony Root, Ginseng, Bupleurum Root, Cinnamon Bark, Atractylodes Lancea Rhizome, Japanese Angelica Root, Pinellia Tuber, Cnidium Rhizome, and Gardenia Fruit.

In the present study, crude drug groups with an ROR >1, a *p* < 0.05 for Fisher’s exact test, and ≥10 reports were also defined as being associated with DILI. Based on this definition, the top three crude drug groups according to ROR were Areca, Glehnia Root and Rhizome, and Safflower > Sappan Wood.

### 2.6. ROR Focusing on Crude Drugs Contained in Kampo Medicines and DILI

The number of reports, RORs, 95% confidence intervals, and *P* values for each crude drug group calculated to determine the association between crude drugs in Kampo medicines and DILI in Section 2.5 are shown in Table S1. Notably, 78 crude drug groups (90 crude drugs) were associated with DILI (ROR >1, *p* < 0.05 for Fisher’s exact test, and ≥10 reports).

The RORs and 95% CIs for each of the crude drugs discussed in Section 2.5 are shown below. In Section 2.5, the analysis results for the crude drug groups indicated by the red dots in the upper right-hand corner (more reports and higher RORs) are as follows: Scutellaria Root (8.63, 7.97–9.35), Gardenia Fruit (8.43, 7.49–9.49), Ginger (6.75, 6.23–7.32), Cnidium Rhizome (6.68, 5.94–7.51), Pinellia Tuber (6.47, 5.87–7.12), Ginseng (6.03, 5.51–6.59), Cinnamon Bark (6.01, 5.47–6.61), Jujube (6.00, 5.49–6.55), Bupleurum Root (5.52, 5.04–6.05), Japanese Angelica Root (5.28, 4.78–5.83), Poria Sclerotium (5.08, 4.65–5.56), Peony Root (4.85, 4.42–5.32), Glycyrrhiza (4.76, 4.46–5.08), and Atractylodes Lancea Rhizome (4.21, 3.8–4.66). In addition, our analysis revealed that the top three crude drug groups associated with DILI in Section 2.5, in order of increasing ROR, were Areca (51.25, 20.33–129.23), Glehnia Root and Rhizome (36.05, 15.84–82.02), and Safflower > Sappan Wood (34.51, 11.87–100.29).

## 3. Discussion

### 3.1. Crude Drugs Associated with DILI

The current study suggests that a number of the crude drugs contained in Kampo medicines are associated with DILI. A systematic literature review was conducted using PubMed, Google Scholar, etc., for identifying all types of reports on crude drugs and DILI, with reference to studies on previously reported adverse reactions to medicinal plants [11].

First, we focused on four crude drugs included in the top three crude drug groups according to their ROR values, which may indicate a high risk of DILI. Accordingly, Areca showed the highest ROR. Consistent with this, some reports showed that Areca nut was associated with liver injury. Areca nut, a toxic substance used extensively worldwide, is known to have several harmful effects on the human body. While they are often associated with oral cancer, studies showed that they affect various organs, including the liver [12,13]. Areca nut contains four alkaloids, namely arecoline, arecaidine, guvacoline, and guvasine, with some studies suggesting the hepatotoxicity of arecoline [14,15]. The present results support the findings of these previous studies. Glehnia Root and Rhizome showed the second-highest ROR. Despite conducting a literature survey on Glehnia Root and Rhizome, we found no information on the related hepatotoxicity. However, when we focused on the coumarin components (imperatorin, psoralen, and bergapten) contained in Glehnia Root and Rhizome, basic studies indicated the hepatotoxicity of psoralen [16,17,18]. The present results may reflect that DILI is caused by psoralen. Considering the strong correlation between Safflower and Sappan Wood, they were considered to be the same crude drug group when calculating RORs, which we found to be the third-highest. Although some reports showed an association between Safflower and acute liver failure [19], many other reports revealed that the components in Safflower reduce hepatotoxicity and exhibit hepatoprotective effects [20,21,22,23]. No reports of hepatotoxicity in Sappan Wood were found. Current studies are in the process of uncovering the pharmacological activities and toxicities of the extracts and chemical constituents of Sappan Wood [24,25,26]. Although no clear information on liver damage was obtained from the previous studies on either crude drug, further research is warranted given that our findings suggested some risk.

Next, among the crude drug groups with a high number of reports and high RORs, 12 crude drugs frequently used in Kampo medicines or associated with DILI in previous studies were highlighted.

The following is a list of crude drugs that were reported to be associated with DILI in previous studies and in the present results. Forsythia Fruit, Schizonepeta Spike, and Saposhnikovia Root and Rhizome were merged into the same crude drug group due to their strong correlation, all of which had a high ROR. Studies showed that Forsythia Fruit exhibits anti-inflammatory effects that prevent fulminant hepatitis while displaying several potential therapeutic effects [27,28]; however, no reports showed an association with liver injury. In contrast, Schizonepeta Spike was cited as a potential cause of hepatotoxicity, and the (+)-menthofuran in Schizonepeta Spike was reported to be involved in hepatotoxicity [29,30,31]. Reports on Saposhnikovia Root and Rhizome in traditional Chinese medicine suggested their involvement in DILI [32,33,34,35]. The present results support the previous findings on Schizonepeta Spike and Saposhnikovia Root and Rhizome but contradict those on Forsythia Fruit. It is possible that the ROR for Forsythia Fruit was also higher given that it was integrated into the same herbal drug group as Schizonepeta Spike and Saposhnikovia Root and Rhizome. Scutellaria Root is also known to cause interstitial lung disease apart from DILI. In Japan, liver injury has long been reported to occur with Kampo medicines containing Scutellaria Root. Therefore, a number of accompanying studies described Kampo medicines with caution in patients with hepatic dysfunction or hepatitis [1,5,6,7]. Gardenia Fruit was reported to be involved in liver dysfunction by increasing aspartate aminotransferase and alanine transaminase, and its main ingredient, geniposide, has been reported to cause liver damage [36,37]. Case reports on the use of Pinellia Tuber in Chinese medicine found that it is associated with liver injury. A case report in Japan describes a patient with acute hepatitis who exhibited jaundice after saireito administration, with a lymphocyte migration inhibition test identifying Pinellia Tuber as the cause [32,38]. For Ginseng, both hepatoprotective and hepatotoxic effects were investigated. Hepatotoxicity is thought to be caused by adverse interactions rather than the direct effects of Ginseng in combination with other drugs such as imatinib [39,40,41]. Bupleurum Root contains saikosaponins, which was reported to cause liver injury when ingested at high doses over a short period. There were also reports of an increased risk of liver injury in hepatitis B patients who used Kampo medicines containing 19 g or more of Bupleurum Root [42,43,44]. Glycyrrhiza contains a hepatoprotective component commonly called glycyrrhizin, which is used in clinical practice. It is known for its various side effects, such as hypokalemia and hypertension due to pseudo-hyperaldosteronism, with other reports also showing that it causes hepatotoxicity [45,46].

Contrary to the results of the present study, the following crude drugs were reported only in studied cases involving suppression of liver injury. Given that these crude drugs are contained in many Kampo medicines, their RORs may be higher owing to pseudo-correlations with other crude drugs. Platycodon Root was reported to exert therapeutic effects on various DILI models [47,48]. Moreover, studies on Atractylodes Rhizome revealed that it exhibits hepatoprotective and antitumor effects against hepatocellular carcinoma cells [49,50]. Ginger was reported to have hepatoprotective effects in a model of DILI [51,52]. Randomized clinical trials were also conducted to investigate the effects of Ginger in patients with nonalcoholic fatty liver [53].

Finally, a report by Arai et al. showed that Artemisia Capillaris Flower could potentially be involved in liver injury [9]. However, its ROR did not significantly exceed 1 in the present study. Other previous studies reported its usefulness in liver dysfunction [54,55,56]. Considering that Artemisia Capillaris Flower is used primarily for treating jaundice, larger-scale clinical studies in humans are expected.

### 3.2. Clinical and Practical Findings

First, the background information of 1701 patients who used Kampo medicines and experienced DILI (Figure 3) is discussed below. Based on the patient demographics, most patients were middle-aged women (in their 50s and 60s). The most common primary diseases were lifestyle-related diseases (hypertension, dyslipidemia, and diabetes mellitus) and diseases common in modern society (constipation, insomnia, gastroesophageal reflux disease, asthma, obesity, and depression). Accordingly, the most commonly used drugs were antihypertensive medications, laxatives, analgesics, stomachic drugs, sleeping pills, antibiotics, and Kampo medicines. Among the top 20 drugs used, the following 8 Kampo medicines each have different uses: bofutsushosan (used for obesity, constipation), saireito (used for gastroenteritis, diarrhea), saikokaryukotsuboreito (used for stress, insomnia), daikenchuto (used for abdominal symptoms), kakkonto (used for common cold, stiff shoulders), orengedokuto (used for dermatitis, stomatitis), hangeshashinto (used for stress, stomach symptoms), and shakuyakukanzoto (used for cramps).

Next, the evaluation of DILI is discussed. In clinical practice, DILI is assessed using tools such as the Roussel Uclaf Causality Assessment Method (RUCAM) and the Digestive Disease Week-Japan (DDW-J) 2004 scale. The corresponding scores are calculated based on the time to onset, previous laboratory data, risk factors (e.g., alcohol consumption, pregnancy, age, and effects of other drugs), presence of potential nondrug-related causes (i.e., diseases that induce liver injury), previous reports on the presence of DILI, eosinophilia, drug lymphocyte stimulation test results, and response to repeated dosing [57,58]. In studies on adverse drug reactions, the extent of these reactions must be determined to take adequate preventive measures. However, the RUCAM and the DDW-J 2004 scale could not be applied to the JADER data used in the present study owing to the lack of laboratory data and some other patient information. Therefore, in the present study, DILI identification was limited to include only the diseases for which adverse events were registered.

Finally, we discussed Kampo medicines in relation to nutrivigilance activities. Since 2010, the French Agency for Food, Environmental and Occupational Health & Safety has been implementing a nutrivigilance plan with the aim of collecting information on any adverse effects caused by the consumption of fortified foods, new foods, novel ingredients, and food supplements; further, the agency is identifying the risk factors for the same [59]. This activity also includes adverse effects caused by Kampo medicines and other herbal products. The safety of Kampo medicines may be overestimated because these medicines have a long history and are considered to have many health benefits. As shown in Figure 3e, bofutsushosan was the most frequently used Kampo drug in patients who experienced DILI. Bofutsushosan is most commonly used to treat obesity and is at risk of being abused for weight loss purposes [60]. Therefore, further studies are needed to determine the mechanisms of action of these drugs and the ways to use them safely and effectively. We hope that the methods of collecting information regarding adverse effects and developing nutrivigilance activities will be improved in the future [61].

### 3.3. Limitations

The current study has several limitations worth noting. We first describe the limitations related to the database that was used. Given that our database contained information on adverse drug reactions based on spontaneous reports, cases were limited to those recognized as adverse drug reactions. Specifically, it is possible that mild side effects were reported occasionally, but severe side effects were reported frequently. This is known as reporting bias, a characteristic of self-reported databases [62]. In the current study, the total number of patients who used each Kampo medicine and the included crude drugs could not be determined; hence, a true assessment of adverse events could not be achieved. As such, we took measures to make the analysis useful by setting a filter for the number of reports and avoiding easy *p* value and ROR comparisons. Second, some JADER data were incomplete, and there may be blank cells, such as missing values, or incorrectly entered letters and numbers. In this study, when missing values for sex and age were found, they were removed and addressed. Third, when multiple drugs are administered, it is difficult to identify the specific cause of the adverse events. Furthermore, fatal adverse events in the JADER database were verified by the PMDA, but other adverse events were based on the reporter’s judgment and could, thus, include not only true but also suspected adverse events.

Nonetheless, the JADER is the largest database comprising voluntary reports of adverse drug reactions in Japan. The adverse drug reaction information obtained from the JADER database is expected to reflect not only pharmacological and pharmacokinetic characteristics but also prescription and usage conditions. Therefore, the JADER is an excellent tool for understanding adverse drug reactions and can be used across several research areas.

The following discussions describe the limitations in Kampo medicine research. Clinically, Kampo medicines are effective when multiple crude drugs interact with each other. In addition, a single crude drug is considered to contain multiple chemical components. Therefore, it is important to make comparisons that consider multiple crude drugs and chemical components. However, given that calculating and evaluating all combinations required an enormous amount of time, this study was limited to ROR calculation after considering multicollinearity, which prevented us from examining the effects of interactions specific to Kampo medicine. Future studies are, therefore, needed to analyze the data using multivariate analysis and machine learning techniques to conduct interaction analysis and compound evaluation.

## 4. Materials and Methods

### 4.1. JADER and Data Management

Published by the PMDA, the JADER is the largest information database in Japan through which trends in the occurrence of adverse drug reactions can be identified. The database can be downloaded from the PMDA website [10]. In this study, we analyzed adverse drug reaction reports submitted to the JADER database from 1 April 2004 to 30 November 2021 (Figure 1). JADER case reports are classified into four tables: DRUG (drug name, causal relationship, etc.), REAC (adverse events, outcome, etc.), DEMO (patient information such as gender, age group, etc.), and HIST (medical history, underlying disease, etc.). In this study, data from the DRUG, REAC, and DEMO tables were used. Drugs detailed in the DRUG table are classified into three categories according to the degree of involvement in adverse events: “suspected drug”, “concomitant drug”, and “interacting drug”, and only data for “suspected drug” were used in this study.

Adverse events in the REAC table are recorded by the preferred terms established by the International Council for Harmonisation of Technical Requirements for Pharmaceuticals for Human Use of Pharmaceutical Terms (Medical Dictionary for Regulatory Activities Japanese version 24.1 (MedDRA/J v24.1)) [63].

Duplicates in the DRUG and REAC tables were removed according to Toriumi et al.’s report [64,65] and combined in the DEMO table using identification numbers. This was used as the report-based dataset. For the report-based dataset, we also determined whether the adverse event was DILI and assigned a DILI flag (dummy variable). In addition, we determined whether the suspected drug was Kampo medicine and assigned a Kampo medicine flag (dummy variable). Simultaneously, we assigned 126 crude drug flags (dummy variables) according to the composition of the Kampo medicine. The dataset was then integrated so that the identification number would be unique. In doing so, priority was given to “applicable” for each assigned flag. Finally, cases with missing gender and age information were excluded in the creation of the patient-based dataset (Figure 1).

### 4.2. Definition of Adverse Events

Adverse events reported in the JADER database that corresponded to DILI were retrieved from the Standardized MedDRA Queries (SMQ) [66], and the following were defined as eligible for analysis. “Drug related hepatic disorders—comprehensive search (SMQ)” included “Cholestasis and jaundice of hepatic origin (SMQ)”, “Liver related investigations, signs and symptoms (SMQ)”, and “Liver-related coagulation and bleeding disturbances (SMQ)”. In addition, “Hepatic failure, fibrosis and cirrhosis and other liver damage-related conditions (SMQ)” and “Hepatitis, non-infectious (SMQ)” were included under “Drug-related hepatic disorders—severe events only (SMQ)”, excluding those events related to malignant and benign tumors not directly associated with DILI. From these SMQs, 228 MedDRA/J v24.1 preferred terms were extracted as DILI (Table S2).

### 4.3. Definition of Crude Drugs Contained in Kampo Medicines

Information on 148 Kampo medicines and 137 crude drugs contained in Kampo medicines was obtained from the Japan Kampo Medicines Manufacturers Association [3]. The composition of the crude drugs used in Japanese Kampo medicines differs depending on the manufacturer. Therefore, the crude drugs contained in Kampo medicines were examined and determined as follows.

If a certain Kampo medicine was produced by only one pharmaceutical company, the composition of that company was adopted. If the same Kampo medicine was produced by more than one manufacturer, the composition of Tsumura & Co., which has the largest sales volume, was used. If the product was not produced by Tsumura & Co., the composition of the company with the earliest start of sales or the composition of the pharmacist with the most experience in clinical practice was consulted to determine the composition (e.g., keishikaryoujutsubuto was assumed to be from OHSUGI Pharmaceutical Co.,Ltd., Osaka, Japan, given that they started selling earlier. Moreover, daisaikotokyodaio was assumed to be from Kotaro Pharmaceutical Co., Ltd., Osaka, Japan). This resulted in a list of 148 medical Kampo medicines and 126 crude drugs included in Kampo medicines, which had been narrowed down from the 137 crude drugs included in the composition (Table S3).

### 4.4. Descriptive Statistics

The number of reports per adverse event was tabulated for the report-based dataset. For the patient-based dataset, the frequencies and percentages were calculated for gender (male, female), age group (age group every 10 years), reporting year, use of Kampo medicines, and occurrence of DILI.

### 4.5. Association between Crude Drugs Contained in Kampo Medicines and DILI

The relationship between the use of each crude drug and the presence or absence of reported DILI was evaluated using the ROR and Fisher’s exact test.

First, the multicollinearity among the dummy variables of the 126 crude drugs contained in the Kampo medicines was examined. Spearman’s correlation coefficient ρ was calculated for all pairs of variables, after which variables with ρ > 0.9 were integrated with each other. Furthermore, among the integrated variables, those with stronger correlations with the objective variable (the dummy variable for DILI) were given priority for integration. Finally, the 126 crude drug variables were integrated into 104 crude drug groups, which were then used as explanatory variables in the analysis.

**Figure 5:**
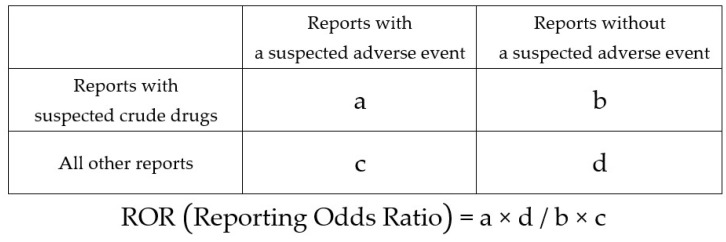
Cross tabulation and formula used to calculate the reporting odds ratio for an adverse event.

Next, a 2 × 2 contingency table of crude drug groups and adverse events was created for all the crude drug groups (Figure 5). This contingency table was then corrected by adding 0.5 to all cells (Haldane Anscombe 1/2 correction) [67,68]. Thereafter, crude drug groups with an ROR > 1 and a *p* value < 0.05 for Fisher’s exact test were determined to be associated with DILI.

At this time, the crude drug groups identified as being associated with DILI included reports of crude drug groups that were statistically significant and had a high ROR but were used in very few cases. Therefore, a lower limit was set on the number of crude drug group reports to detect more reliable signals. This method was adopted given that filtering using only *p* value has two disadvantages. First, if a significance level of *p* value < 0.05 was satisfied, no statistically useful information could be obtained from further *p* value comparisons. In such cases, it is necessary to utilize RORs instead of *p* values. Second, if filtering is performed according to *p* value, there is a possibility that information on frequently reported crude drug groups may be overlooked. Therefore, the lower limit was defined by the value of a + b in the contingency table (Figure 5) (i.e., the total number of reports on using a particular crude drug). With reference to reports on signal detection [69,70,71], the lower limit was set to ≥10 cases to select the crude drugs associated with DILI.

In addition, a volcano plot consisting of the RORs and *p* values of all the crude drug groups was created (Figure 4). The natural logarithm of the ROR (Ln (ROR)) and the ordinary logarithm of the *p* value converted to negative (−Log (*p* value)) were used to create the scatterplot. The plot diagram, called a volcano plot, has been frequently used in bioinformatics and adverse drug reaction studies to identify trends in gene expression and adverse drug reactions [72,73]. The number of reports, RORs, 95% confidence intervals, and *p* values for each crude drug group are then shown in Table S1.

### 4.6. Statistical Analysis Software

Data processing was performed using JMP Pro 16.0 (SAS, Cary, NC, USA) and R version 4.2.1. All statistical analyses were also performed using JMP Pro 16.0.

## 5. Conclusions

Through the JADER, a large dataset from Japan, we investigated the crude drugs contained in Kampo medicines associated with DILI through a comprehensive analysis of the adverse reaction reports involving 148 Kampo medicines prescribed throughout Japan. Accordingly, the current study found that DILI was the most frequently reported and important side effect that should be addressed. Second, we were able to identify the crude drugs contained in the Kampo medicines that were associated with DILI. Further validation and clarification of the underlying mechanisms are expected to contribute to the appropriate management of the adverse drug reactions caused by Kampo medicines and crude drugs.

## Figures and Tables

**Table 1:** Ranking of the top 50 adverse events in the report-based dataset (N = 1,865,069 records).

Rank	Adverse Event	Reporting Times *
1	Interstitial lung disease	51,347
2	Hepatic function abnormal	33,663
3	Platelet count	32,904
4	Neutrophil count decreased	27,409
5	Pyrexia	25,662
6	Pneumonia	25,457
7	White blood cell count decreased	25,134
8	Anemia	22,024
9	Neutropenia	21,246
10	Liver disorder	20,746
11	Anaphylactic shock	20,616
12	Renal impairment	20,069
13	Febrile neutropenia	19,453
14	Rash	17,950
15	Diarrhea	17,246
16	Drug eruption	15,883
17	Acute kidney injury	14,622
18	Decreased appetite	14,227
19	Anaphylactic reaction	12,298
20	Pancytopenia	12,078
21	Stevens–Johnson syndrome	12,020
22	Thrombocytopenia	12,012
23	Rhabdomyolysis	11,998
24	Nausea	11,839
25	Blood pressure decreased	11,615
26	Cerebral infarction	10,860
27	Cardiac failure	10,824
28	Myelosuppression	10,552
29	Sepsis	10,549
30	Erythema multiforme	10,516
31	Hypoglycemia	10,309
32	Vomiting	10,190
33	Hemoglobin decreased	9930
34	Altered state of consciousness	9907
35	Seizure	9624
36	Drug-induced liver injury	9546
37	Death	9010
38	Leukopenia	8875
39	Fatigue	8785
40	Toxic epidermal necrolysis	8152
41	Cerebral hemorrhage	8063
42	Loss of consciousness	7890
43	Erythema	7711
44	Dyspnea	7671
45	Neuroleptic malignant syndrome	7597
46	Drug reaction with eosinophilia and systemic symptoms	7589
47	Pneumocystis jirovecii pneumonia	7405
48	Disseminated intravascular coagulation	7301
49	Agranulocytosis	7066
50	Shock	6741

* “Reporting times” indicates the number of times each adverse event was reported for the suspected drug.

## Data Availability

Data are contained within the article and the Supplementary Materials.

## Supplementary Materials

The following supporting information can be downloaded at https://www.mdpi.com/article/10.3390/ph16050678/s1. Table S1: Reporting odds ratio for each crude drug group (sorted by descending reporting odds ratio); Table S2: SMQ defined as drug-induced liver injury; Table S3: List of Kampo medicines for prescription in Japan, the crude drugs they contain, and the pharmaceutical companies referenced.
